# Supplementary material for: Prefoldin 5 is a microtubule-associated protein that suppresses Tau aggregation and neurotoxicity
Source: eLife. 2026 Jan 14;13:RP104691. doi: 10.7554/eLife.104691 (PMC12803513; doi:10.7554/eLife.104691)
Supplement: Figure 2—source data 2. [file elife-104691-fig2-data2.zip › Figure 2-Source data 2/Figure 2-Source data 2.pdf]

Ace-tubulin

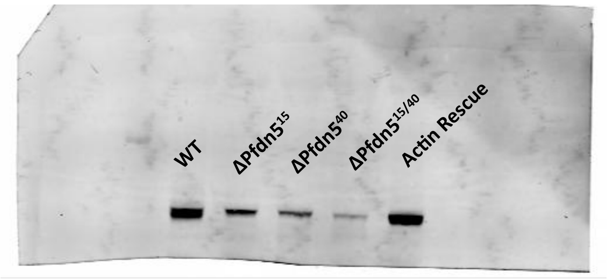

α-Tubulin

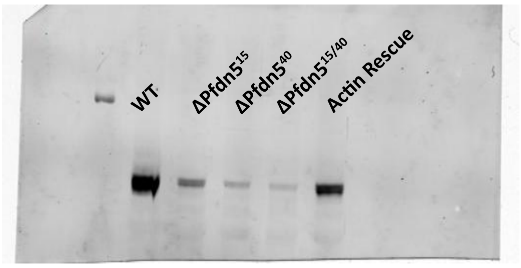

Ran

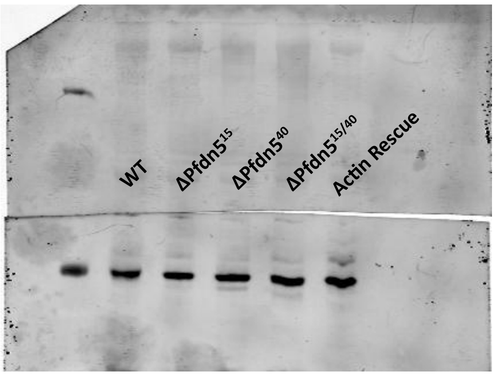

β-actin

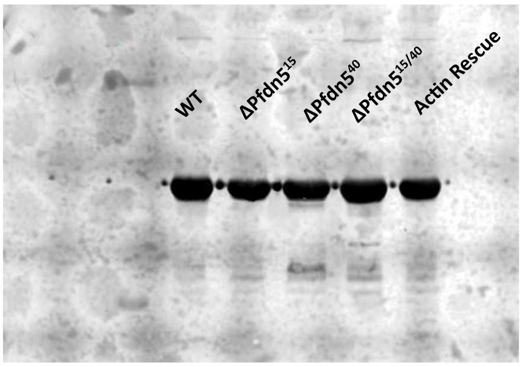

β-Tubulin

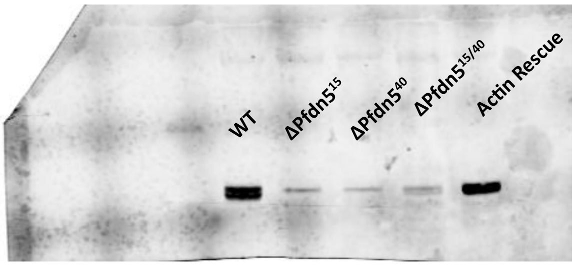

Figure 2-source data 2. Original membranes corresponding to Figure 2, panel G.
