# Supplementary material for: Prefoldin 5 is a microtubule-associated protein that suppresses Tau aggregation and neurotoxicity
Source: eLife. 2026 Jan 14;13:RP104691. doi: 10.7554/eLife.104691 (PMC12803513; doi:10.7554/eLife.104691)
Supplement: Figure 3—source data 2. [file elife-104691-fig3-data2.zip › Figure 3-Source data 2/Figure 3-Source data 2.pdf]

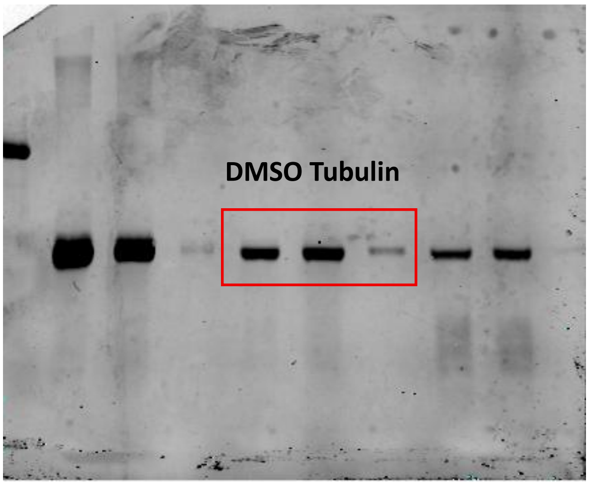
DMSO Tubulin

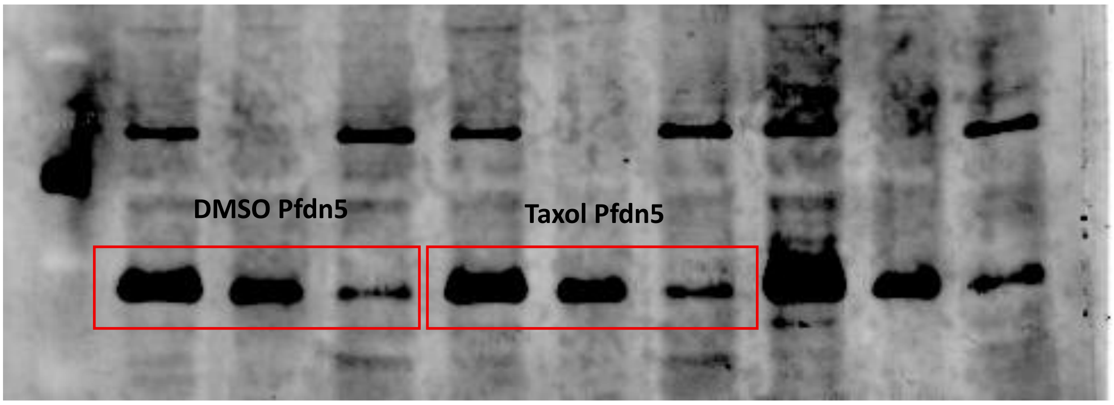
DMSO Pfdn5
Taxol Pfdn5

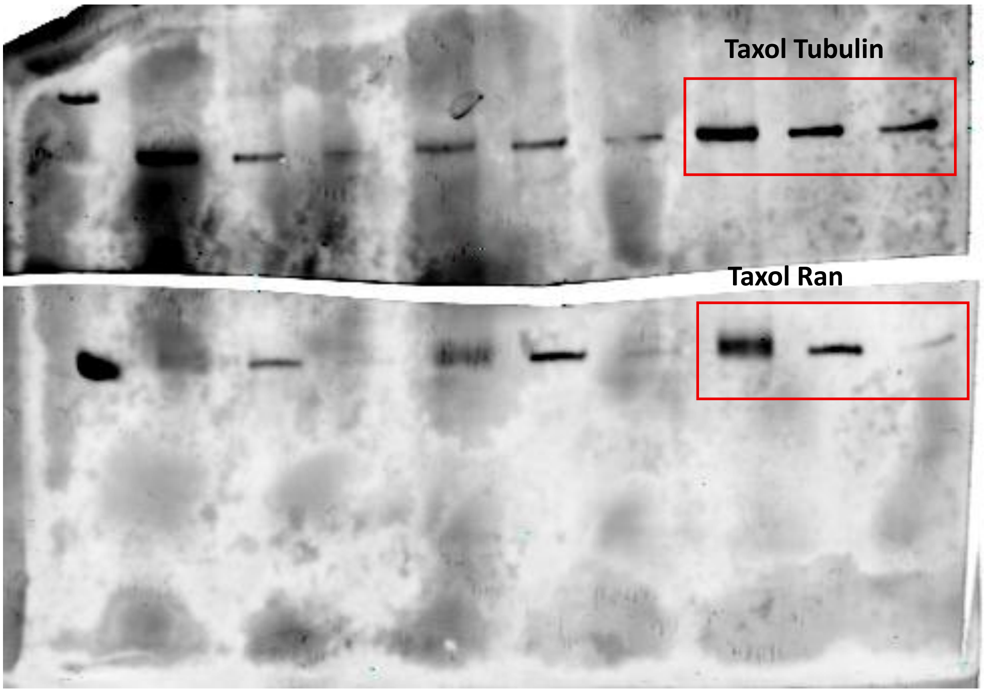
Taxol Tubulin
Taxol Ran

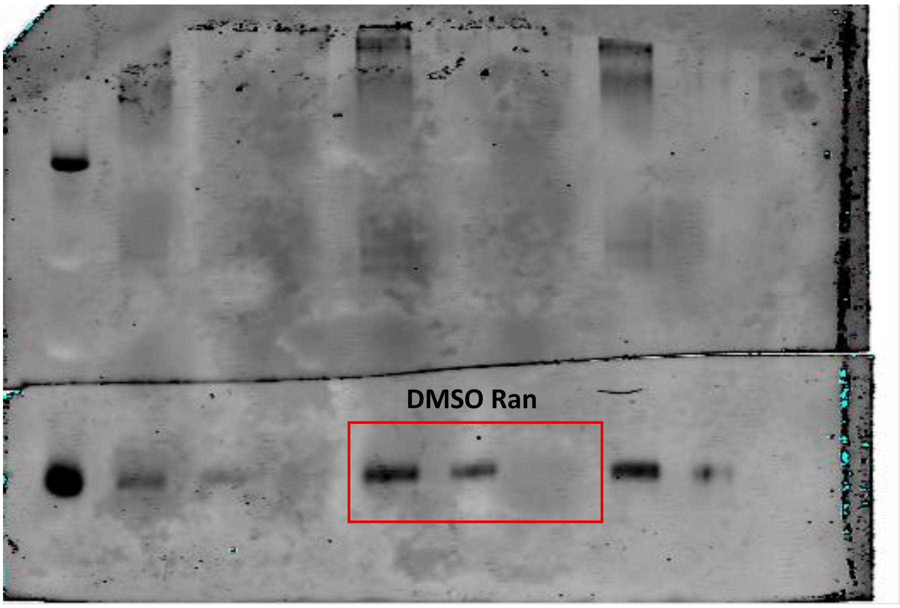
DMSO Ran

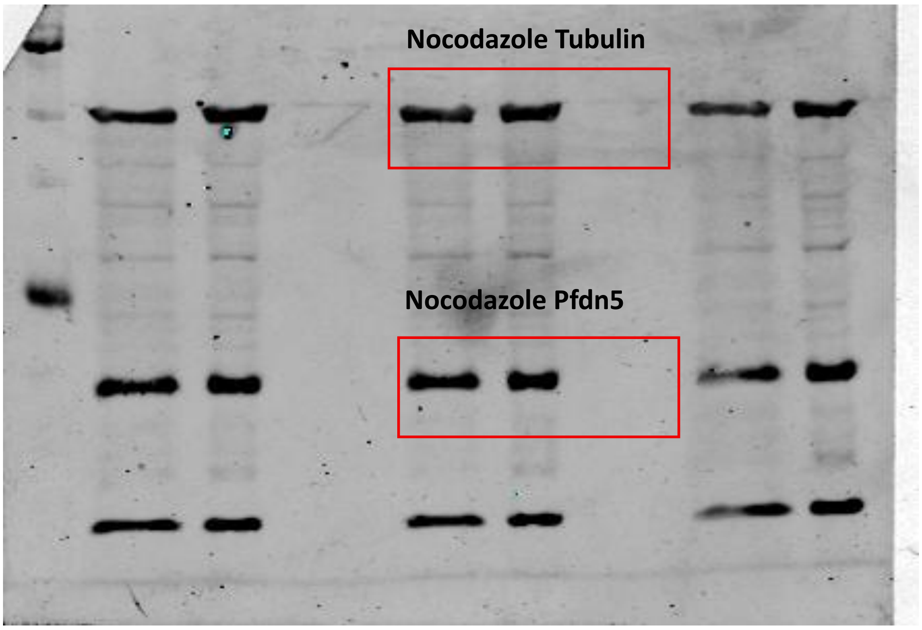
Nocodazole Tubulin
Nocodazole Pfdn5

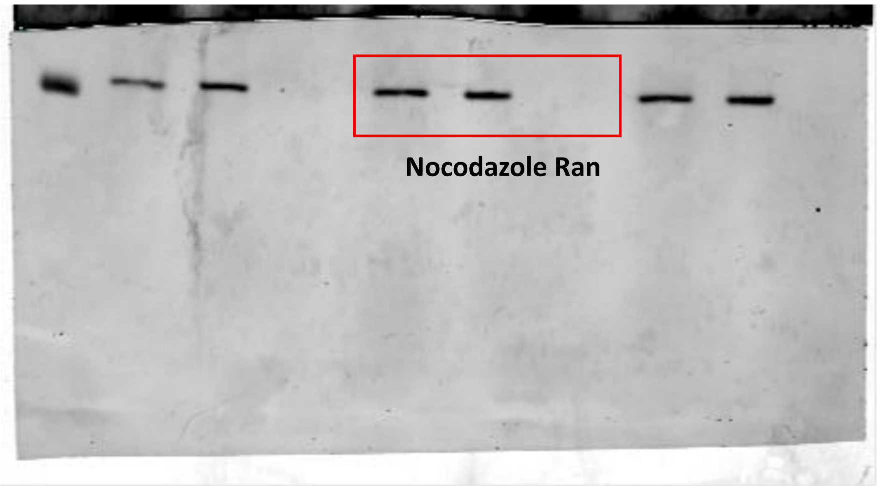

Figure 3-source data 2. Original membranes corresponding to Figure 3, panel F. Relevant bands are labelled and marked in red boxes. Remaining or unmarked bands are of the irrelevant samples.
