# Supplementary figures and images for: Prefoldin 5 is a microtubule-associated protein that suppresses Tau aggregation and neurotoxicity
Source: eLife. 2026 Jan 14;13:RP104691. doi: 10.7554/eLife.104691 (PMC12803513; doi:10.7554/eLife.104691)

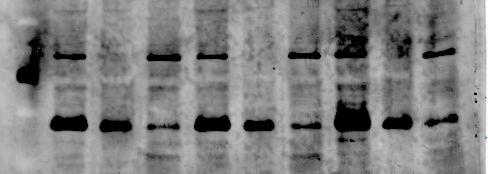

Supplement: Figure 3—source data 3. [file elife-104691-fig3-data3.zip › Figure 3-Source data 3/Pfdn5_DMSO(lane 2-4) and taxol (lane 5-7).tif]

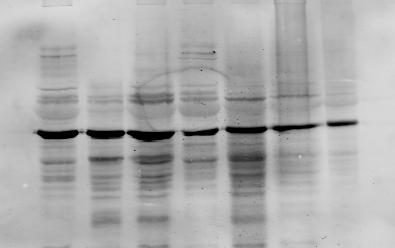

Supplement: Figure 3—figure supplement 1—source data 3. [file elife-104691-fig3-figsupp1-data3.zip › Figure 3-figure supplement 1-source data 3/GAPDH in Pfdn5 heterzygous (Lane2-4).tif]

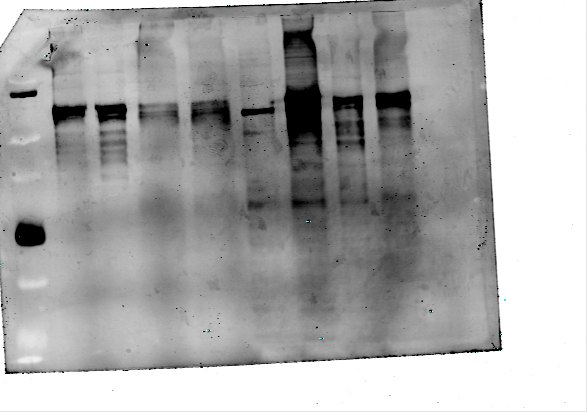

Supplement: Figure 5—figure supplement 2—source data 3. [file elife-104691-fig5-figsupp2-data3.zip › Figure 5-figure supplement 2-source data 3/Tau solubility assay D5D8N in pellet (lane 4-5).tif]

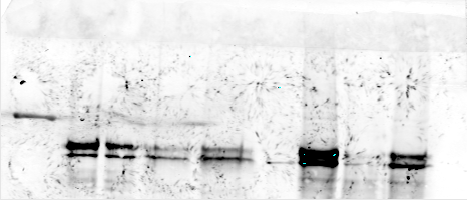

Supplement: Figure 5—figure supplement 2—source data 3. [file elife-104691-fig5-figsupp2-data3.zip › Figure 5-figure supplement 2-source data 3/Tau solubility assay D5D8N in supernatant (lane 2-3).tif]

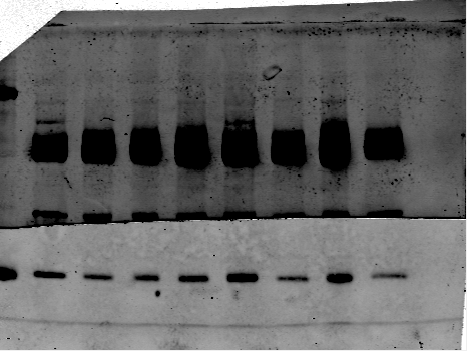

Supplement: Figure 8—source data 3. [file elife-104691-fig8-data3.zip › Figure 8-Source data 3/Ran_lower cut blot (lane 2-4).tif]

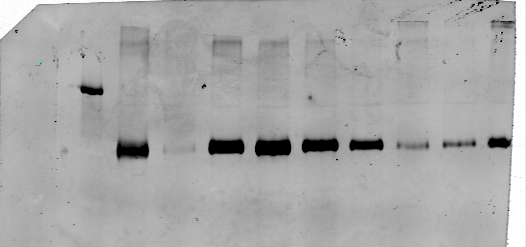

Supplement: Figure 8—figure supplement 1—source data 3. [file elife-104691-fig8-figsupp1-data3.zip › Figure 8-figure supplement 1-source data 3/alpha tubulin_reprobed (lane 2-5).tif]
