# Supplementary material for: Prefoldin 5 is a microtubule-associated protein that suppresses Tau aggregation and neurotoxicity
Source: eLife. 2026 Jan 14;13:RP104691. doi: 10.7554/eLife.104691 (PMC12803513; doi:10.7554/eLife.104691)
Supplement: Figure 3—figure supplement 1—source data 2. [file elife-104691-fig3-figsupp1-data2.zip › Figure 3-figure supplement 1-source data 2/Figure 3-figure supplement 1-source data 2.pdf]

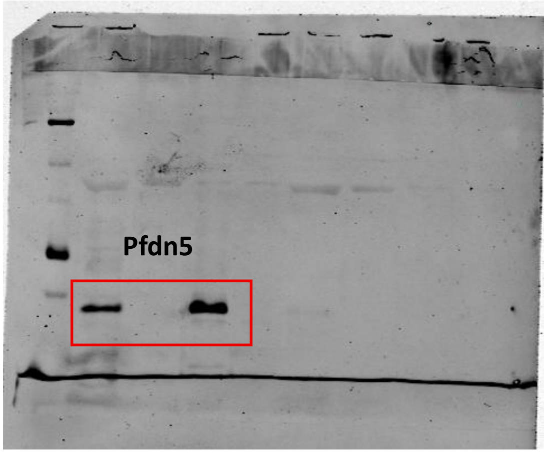
Pfdn5

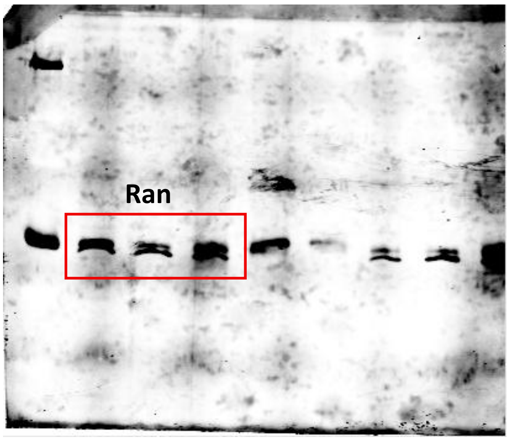
Ran

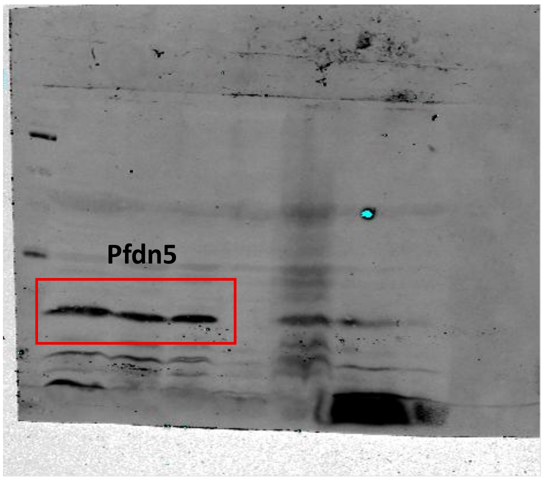
Pfdn5

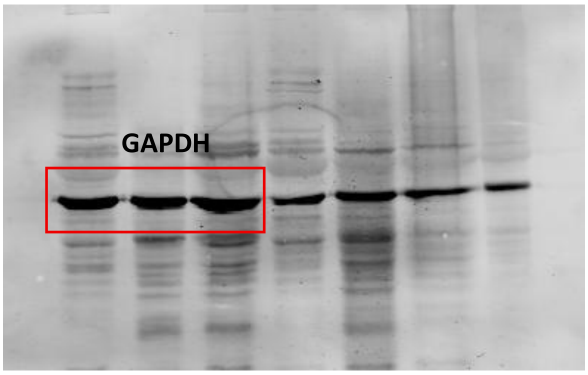
GAPDH

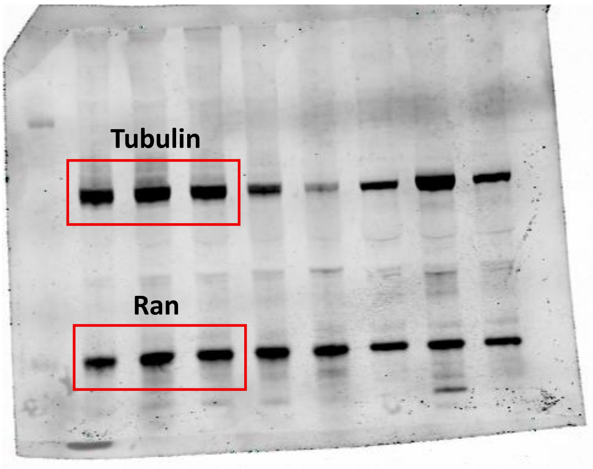
Tubulin
Ran

Figure 3-Figure supplement 1-source data 2. Original membranes corresponding to Figure 3-Figure supplement 1, panel A and F. Relevant bands are labelled and marked in red boxes. Remaining or unmarked bands are of the irrelevant samples. Red-marked boxes of the first two blots indicate the following genotypes (lane 1: Control, lane 2: ΔPfdn5$^{15/40}$, Lane 3: Actin Rescue). Red-marked boxes of the last three blots indicate the following genotypes (lane 1: Control, lane 2: ΔPfdn5$^{15/+}$, Lane 3: ΔPfdn5$^{40/+}$).
