# Supplementary material for: Prefoldin 5 is a microtubule-associated protein that suppresses Tau aggregation and neurotoxicity
Source: eLife. 2026 Jan 14;13:RP104691. doi: 10.7554/eLife.104691 (PMC12803513; doi:10.7554/eLife.104691)
Supplement: Figure 6—source data 2. [file elife-104691-fig6-data2.zip › Figure 6-Source data 2/Figure 6-Source data 2.pdf]

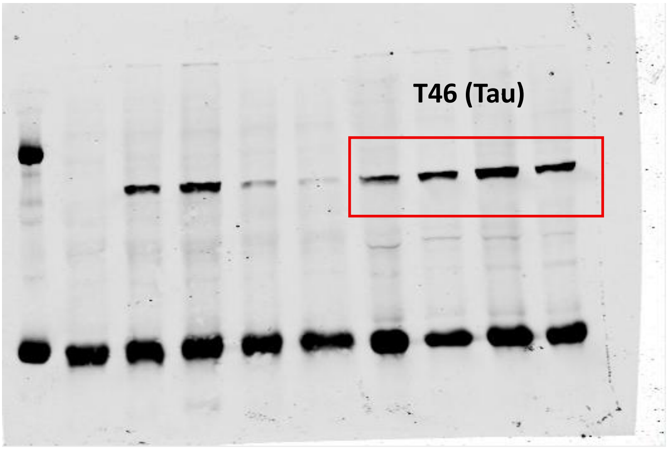

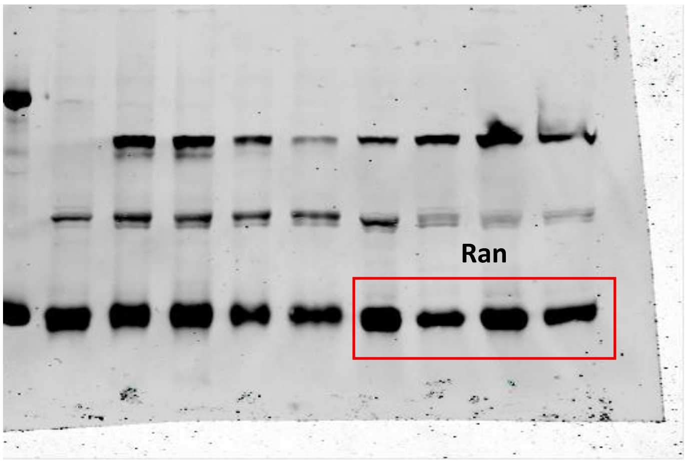

Figure 6-source data 2. Original membranes corresponding to Figure 6, panel O. Relevant bands are labelled and marked in red boxes. Remaining or unmarked bands are of the irrelevant samples. Red-marked boxes indicate the following genotypes (lane 1: Elav>UAS-hTau^{V337M}, lane 2: Elav>UAS-hTau^{V337M}; UAS-GFP, Lane 3: Elav>UAS-hTau^{V337M}; UAS-Pfdn5, Lane 4: Elav>UAS-hTau^{V337M}; UAS-Pfdn6).
