# Supplementary material for: Prefoldin 5 is a microtubule-associated protein that suppresses Tau aggregation and neurotoxicity
Source: eLife. 2026 Jan 14;13:RP104691. doi: 10.7554/eLife.104691 (PMC12803513; doi:10.7554/eLife.104691)
Supplement: Figure 8—figure supplement 1—source data 2. [file elife-104691-fig8-figsupp1-data2.zip › Figure 8-figure supplement 1-source data 2/Figure 8-figure supplement 1-source data 2.pdf]

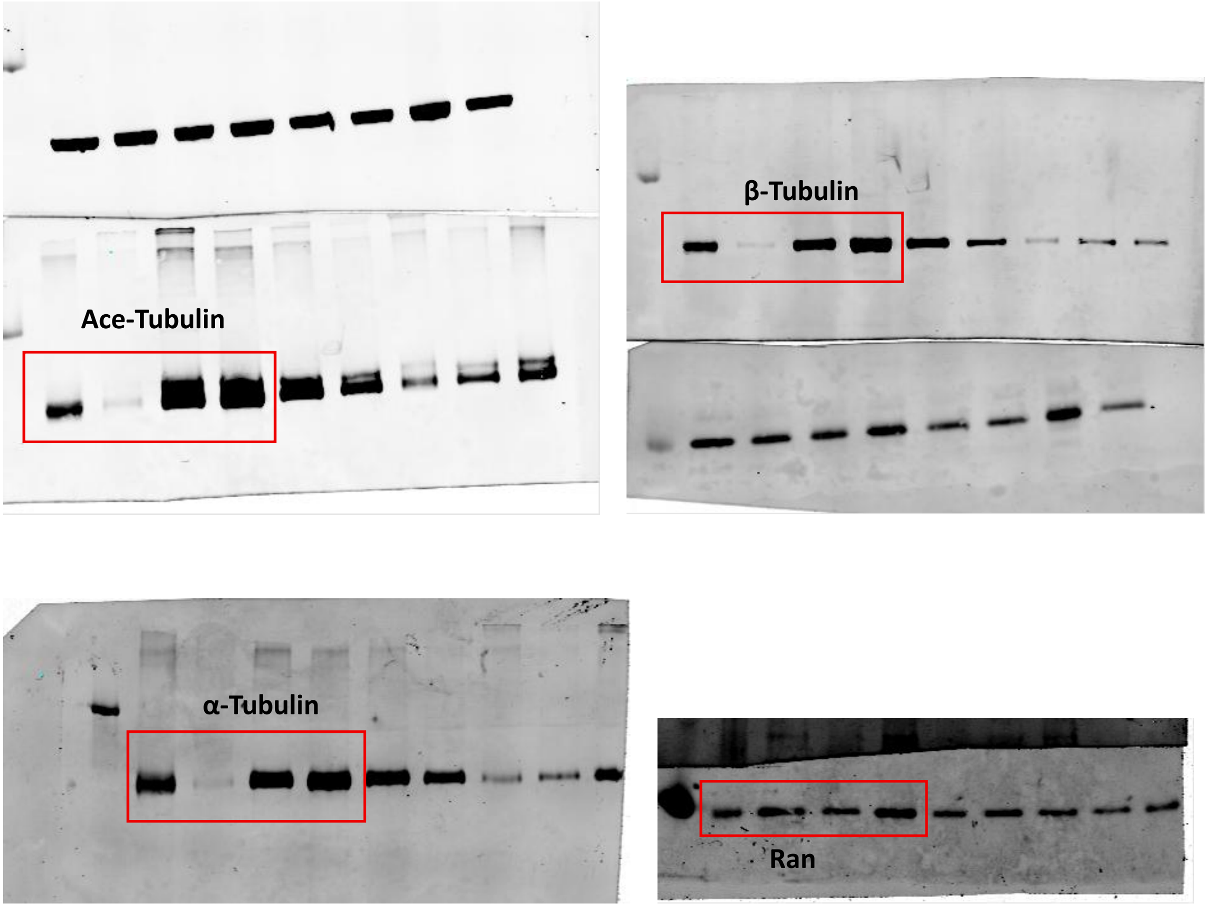

Figure 8-Figure supplement 1-source data 2. Original membranes corresponding to Figure 8-Figure supplement 1, panel A. Relevant bands are labelled and marked in red boxes. Remaining or unmarked bands are of the irrelevant samples. Red-marked boxes indicate the following genotypes (lane 1: Control, lane 2: ΔPfdn5$^{15/40}$, lane 3: Elav>UAS-α-Tubulin; ΔPfdn5$^{15/40}$, lane 4: Elav>UAS-Pfdn5; ΔPfdn5$^{15/40}$). Blots are cut and staining with the labelled antibodies.
